# Validity and Reliability of a Food Frequency Questionnaire (NFFQ-Kids) to Assess Food Consumption Based on the Nova Classification in Southern Italian Children and Adolescents

**DOI:** 10.3390/nu17233751

**Published:** 2025-11-28

**Authors:** Nadia Paladino, Giuseppe Di Costanzo, Emilia Ruggiero, Augusto Di Castelnuovo, Marika Dello Russo, Annarita Formisano, Fabio Lauria, Walter Currenti, Fabio Galvano, Giuseppe Grosso, Licia Iacoviello, Marialaura Bonaccio

**Affiliations:** 1 Department of Biomedical and Biotechnological Sciences, University of Catania, 95124 Catania, Italy; nadia.paladino@phd.unict.it (N.P.); walter.currenti@unict.it (W.C.); giuseppe.grosso@unict.it (G.G.); 2 Research Unit of Epidemiology and Prevention, Istituto Neurologico Mediterraneo IRCCS NEUROMED, 86077 Pozzilli, Italy; giuseppe.dicostanzo@moli-sani.org (G.D.C.); augusto.dicastelnuovo@moli-sani.org (A.D.C.); licia.iacoviello@moli-sani.org (L.I.); marialaura.bonaccio@moli-sani.org (M.B.); 3 Institute of Food Sciences, Consiglio Nazionale delle Ricerche, 83100 Avellino, Italy; marika.dellorusso@isa.cnr.it (M.D.R.); annarita.formisano@isa.cnr.it (A.F.); fabio.lauria@isa.cnr.it (F.L.); 4 College of Health Science, Princess Nourah Bint Abdulrahman University, Riyad 11564, Saudi Arabia; 5 Center for Human Nutrition and Mediterranean Foods (NUTREA), University of Catania, 95124 Catania, Italy; 6 Department of Medicine and Surgery, LUM University, 70010 Casamassima, Italy

**Keywords:** food frequency questionnaire, Nova classification, ultra-processed foods, validity, reliability, children/adolescents

## Abstract

**Background/Objectives**: There is a lack of food frequency questionnaires (FFQs) specifically designed to assess food consumption based on processing levels in younger populations. This study evaluates the validity and the reliability of a 107-item FFQ (NFFQ-Kids) in estimating the intake (g/day) and the weight and energy ratios of Nova groups in Italian children and adolescents aged 2–18 years. **Methods**: The NFFQ-Kids was administered twice (T0 and T1), with a four-week interval. A total of 73 participants completed the questionnaire at T0, and 53 completed it at T1. Participants were selected from the ICARO Study (December 2023–April 2024), a web-based cohort study in Southern Italy. Questionnaire validity was assessed by comparison with a 3-day food record (two weekdays and one weekend day) collected between the two NFFQ-Kids administrations. Test–retest reliability was evaluated to assess the consistency of the questionnaire over time. **Results**: A moderate correlation was found between the NFFQ-Kids and the 3-day food record for the energy ratio of ultra-processed foods (UPFs) (r = 0.48; *p* < 0.001; ICC = 0.46; 95% CI 0.29–0.64) and the weight ratio for both unprocessed or minimally processed foods (r = 0.49; *p* < 0.001; ICC = 0.50; 95%CI 0.33–0.66) and ultra-processed foods (UPFs) (r = 0.58; *p* < 0.001; ICC = 0.58; 95%CI 0.42–0.72). Overall, the NFFQ-Kids showed good test–retest reliability across all Nova group intakes, total food, and relative energy and weight ratios of UPFs (r = 0.71, ICC = 0.67; and r = 0.79, ICC = 0.67, respectively), indicating stable measurements over time. **Conclusions**: The NFFQ-Kids demonstrated acceptable validity and good reliability, proving useful for assessing food intake by processing level in Italian youth.

## 1. Introduction

In recent years, there has been a progressive shift from traditional diets toward Western diets, which are characterized by a higher consumption of ultra-processed foods (UPFs) [1]. The Nova classification categorizes foods according to the extent and purpose of industrial processing into four groups: (i) unprocessed or minimally processed foods (MPFs), (ii) processed culinary ingredients (PCI), (iii) processed foods (PF), and (iv) UPFs, which are formulations of ingredients that results from a multitude of industrial processes to which foods are subjected in part or completely, for example, through the addition of food additives [2]. Several aspects make UPFs the easiest choice for adults and children: they are convenient, hyper-palatable, ready-to-use, and have a long shelf-life, due to the abundance of specific food additives [3]. Nonetheless, UPFs frequently exhibit critical nutritional deficiencies due to the scarcity of micronutrients, macronutrients, and fiber, in favor of an excess of added sugars, sodium, and saturated and trans fats [4]. It has also been shown that higher consumption of UPFs is negatively associated with diet quality [4]. Growing epidemiological evidence from robust cohorts worldwide reported an association between higher UPF intake and detrimental effects on human health [5], such as increased cardiometabolic risk [6], dementia [7], increased risk of certain types of cancer (i.e., colorectal, breast, and pancreatic cancer) [8], and all-cause mortality [5]. Also, children and adolescents with an elevated consumption of UPFs experience negative conditions, such as obesity, impaired body composition, and other cardiometabolic comorbidities, including dyslipidemia, altered blood sugar and blood pressure levels [9]. Furthermore, a recent cross-sectional study showed a significant association between UPF intake and allergy in a cohort of children and adolescents [10], although other studies reported a non-significant relationship [11].

However, most of the evidence on the health effects of food processing in adults comes from studies that have used food frequency questionnaires (FFQs) not specifically designed to assess food intake according to the Nova classification [12,13]. Similarly, there is a lack of validated, age-appropriate tools specifically designed to evaluate food intake according to processing levels in children and adolescents. This gap is particularly relevant for Italian children and adolescents, where rising UPF consumption and declining adherence to the Mediterranean diet highlight the need for locally validated tools [14]. With around 30% of children overweight or obese, especially in southern regions [15], studying dietary patterns by food processing can provide relevant evidence to inform targeted public health strategies.

Given the limited availability of tools specifically adapted for measuring UPF intake among children using the Nova framework, the development of an adapted FFQ is warranted to better assess exposure to food processing in this population. To address this need, the present study aimed to evaluate the relative validity and the reliability of a novel FFQ (NFFQ-Kids) specifically designed to estimate dietary intake according to the Nova classification in Southern Italian children and adolescents. We hypothesized that the NFFQ-Kids would show moderate-to-high correlation with a 3-day food record.

## 2. Materials and Methods

### 2.1. Participants

Ninety-nine families participating in the ICARO Study [16], an ongoing web-based cohort study in Southern Italy, were invited to take part in the validation study conducted between December 2023–April 2024. These families included 145 eligible children and adolescents aged 2–18 years. Of these, 82 participants returned the 3-day food record (FR), of which six were incomplete and three reported implausible energy intakes. Consequently, 73 participants with complete and plausible 3-day FRs were retained and included in the analyses. The overall response rate (56.6%) is comparable to those reported in pediatric studies [17].

The sample size was determined pragmatically, based on comparable validation studies in children and adults [18,19] and was considered adequate for assessing the reliability and validity of the NFFQ-Kids. Among the 73 included participants, 43 (58.9%) were aged 2–11 years, and 30 (41.1%) were aged 12–18 years, following commonly used age groupings in pediatric research [20]; moreover, 53 (72.6%) also completed the follow-up questionnaire for test–retest reliability, a retention rate consistent with longitudinal web-based studies [21].

Participants comprised boys and girls residing in Southern Italy (i.e., Abruzzo, Campania, and Molise regions). The study was conducted according to the guidelines of the Declaration of Helsinki and was granted ethical approval by the Ethics Committee of the IRCCS Neuromed, Pozzilli, Italy. For participants under 18 years of age, informed consent was obtained online from their parents prior to questionnaire administration.

### 2.2. Study Design and Data Collection

Participants were recruited through multimedia campaigns, school networks, healthcare professionals, and public meetings. One parent per household completed questionnaires for children under 14, while participants aged > 14 years completed them independently or with assistance. The 14-year cut-off for parental reporting was chosen to minimize reporting bias, as children under this age may have limited ability to accurately self-report dietary intake [22]. Once the informed consent was completed and re-sent, participants were formally included in the project. Participants provided socio-demographic data (i.e., name, surname, age, sex, place of birth, weight, height), presence of allergies and/or intolerances (i.e., celiac disease, lactose intolerance), presence of diseases (i.e., type 1 diabetes), possible nutritional intervention, type of childbirth, and infant feeding type of their children. Participants received detailed written instructions from trained staff and were committed to the completion of NFFQ-Kids and a 3-day FR of children < 14 years old, while adolescents ≥ 14 were allowed to complete them by themselves. The FFQ was collected on 2 appointments, two to four weeks apart. The length of time was selected to minimize recall bias while being brief enough to avoid alterations in the characteristics under investigation. Participants were instructed to complete a 3-day FR. 

In order to prevent the personal identity of respondents, the data was anonymized and examined in aggregate form.

### 2.3. The NFFQ-Kids

The NFFQ-Kids is a modified version of the NFFQ for the Italian adult population [18] and was adapted to collect dietary intakes and the level of processing of the diet of Italian children aged ≥2 and ≤18 years over the past 3 months. The NFFQ-Kids includes an integrated list of 107 items (full description available in Supplementary Materials), which incorporates additional food items specifically selected to better capture the dietary habits of children and adolescents, beyond those in the original NFFQ. The selection of items was guided by their relevance to the pediatric population rather than solely by consumption frequency or nutritional contribution. All food items were classified according to the Nova system and reviewed by two trained researchers, with any discrepancies resolved through discussion until full consensus was reached, ensuring consistent and reliable classification.

### 2.4. The 3-Day Food Record (FR)

A 3-day FR was selected as a reference method, and was completed on three non-consecutive days, including two weekdays and one weekend day, between the two FFQ assessments to ensure a balanced representation of dietary intake. The completeness of the 3-day FR was checked by trained staff for missing meals, implausible portions, and inconsistencies. For homemade recipes participants were asked to indicate every single ingredient and the relative measure in grams or milliliters. Nutritional data were carefully analyzed and then checked for errors. Energy intake was estimated by the software application Metadieta Software EDU 4.5 (METEDA S.r.l., Roma, Italy), which is linked to the Italian database of food composition [23]. After collection, the FRs were reviewed by trained personnel for quality and completeness of nutritional information. Only FRs containing clear and detailed descriptions of the food items and portion sizes consumed were included in the analysis; in cases of minor uncertainties, clarification was sought directly from the participants or their parents.

In addition, all foods and beverages were classified using all available information (product name, brand name, and/or ingredients when feasible) based on the Nova categories used for the food items listed in the NFFQ-Kids. Portion sizes were multiplied by the chosen frequency response in both the 3-day FR and the NFFQ-Kids.

### 2.5. Statistical Analysis

To estimate daily intake (g/day) for each Nova group, the food groups included in each Nova category were summed. Subsequently, the energy and weight ratios were calculated as the proportion (%) of each Nova group relative to the total energy intake and total weight (g/day) of foods consumed, respectively. Validity and reproducibility of the NFFQ-Kids were evaluated in the whole sample, by sex (males and females), and age groups (2–11 and 12–18 years). Pearson correlation coefficients (r) were calculated to test the temporal stability of the FFQ by establishing the correlation of energy and weight ratios between FFQs at T0 at baseline and T1 after 2–4 weeks, a time interval chosen to minimize recall bias while avoiding meaningful changes in dietary habits. Relative validity was tested by assessing the correlation of energy and weight ratios between the NFFQ-Kids and 3-day FR as Pearson correlation coefficients. Furthermore, Bland–Altman plots with upper and lower limits of agreement, supported by paired *t*-tests, were generated to assess agreement and systematic differences between the NFFQ-Kids and the 3-day FR. The data analysis was generated using SAS/STAT software, version 9.4 (SAS Institute Inc., Cary, NC, USA).

## 3. Results

### 3.1. Study Population

The whole sample comprised 73 participants who completed both the NFFQ-Kids and the 3-day FR at T0; of these, 53 (73%) also completed the NFFQ-Kids at T1 and were included in the test–retest reliability analysis. The average age across the sample was 10.6 years, with a slightly higher proportion of males among younger children (46.5%) than adolescents (36.7%). Most participants resided in urban areas (79.5%), particularly in the older age group (86.7%). Food allergies and special diets were more frequently reported among adolescents (Table 1).

### 3.2. Relative Questionnaire Validity

The validity of the NFFQ-Kids was assessed in a sample of 73 subjects who correctly completed both the NFFQ-Kids and a 3-day FR. Validity results for the entire sample are reported in Table 2.

**Figure 1:**
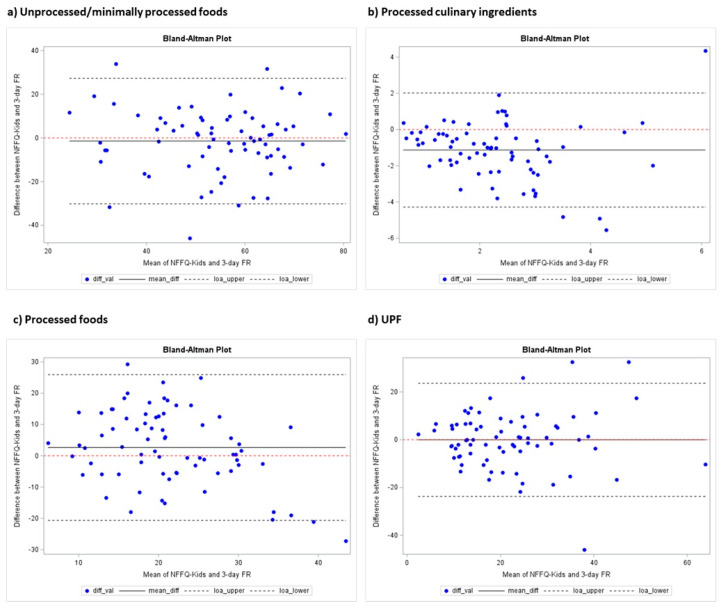
Bland–Altman plots comparing the weight ratios of each Nova food group between the NFFQ-Kids and the 3-day food records. The solid line represents the mean difference between methods, and the dashed lines indicate the 95% limits of agreement.

The Bland–Altman plots show that the NFFQ-Kids has acceptable agreement with the 3-day FR. It shows good average agreement for UPF (Figure 1d, paired *t*-test *p* = 0.97), unprocessed or minimally processed foods (Figure 1a, paired *t*-test *p* = 0.40), and processed foods (Figure 1c, paired *t*-test *p* = 0.063) with small, stable differences. Overall, the NFFQ-Kids is valid at the group level but shows some individual variability.

Among children aged between 2 and 11 years (*n* = 43), a moderate correlation was also found for both the energy ratio (r = 0.48; *p* < 0.001; ICC = 0.50; 95%CI 0.29–0.71) and the weight ratio of UPFs (r = 0.67; *p* < 0.001; ICC = 0.70; 95%CI 0.53–0.83) (Supplementary Table S1). In adolescents aged 12–18 years (*n* = 30), a fair-to-moderate correlation was observed for the energy ratio (r = 0.47; *p* < 0.001; ICC = 0.40; 95%CI 0.16–0.70) and the weight ratio of UPFs (r = 0.41; *p* = 0.026; ICC = 0.41; 95%CI 0.17–0.71) (Supplementary Table S2).

The results of analyses stratified by sex are presented in Supplementary Tables S3 and S4.

Consistent with previous findings, a moderate correlation was observed between the NFFQ-Kids and the 3-day FR in females (*n* = 42) for both the energy ratio (r = 0.60; *p* < 0.001; ICC = 0.47; 95%CI 0.25–0.69) and the weight ratio of UPFs (r = 0.59; *p* < 0.001; ICC = 0.54; 95%CI 0.33–0.74) (Supplementary Table S3).

In males (*n* = 31), a moderate correlation was also found for the weight ratio of UPFs (r = 0.53; *p* < 0.001; ICC = 0.31; 95%CI 0.09–0.67), while the correlation for the energy ratio was fair (r = 0.36; *p* = 0.044; ICC = 0.73; 95%CI 0.54–0.86) (Supplementary Table S4).

Despite these correlations, the NFFQ-Kids showed a tendency to overestimate food intake, particularly for both UPFs and MPFs, when compared to the 3-day FR.

We observed consistently higher correlations and agreement across all Nova categories in the parent-assisted group (*n* = 41) (Supplementary Table S5) compared with the self-administered group (*n* = 32) (Supplementary Table S6).

### 3.3. Test–Retest Reliability

Test–retest reliability was established on a sub-sample composed of 53 participants who adequately completed both FFQs (Table 3). The 20 participants who were not included in the test–retest analysis were older (14.9 ± 4.5 vs. 9.0 ± 4.4 years, *p* < 0.0001) and more frequently resided in urban areas (*p* = 0.043). No differences were observed in sex, presence of food allergies or intolerances, or adherence to a special diet.

The data collected during both assessment periods showed overall good test–retest reliability for all Nova groups’ intakes, total food and energy intakes, and the relative energy and weight ratios (0.42 < r < 0.79; 0.33 < ICC < 0.67, with significant *p* values for all). In this instance, stratification by age and sex was also conducted, as illustrated in Supplementary Tables S7–S10. The results suggest a good consistency of the measure over time, especially in children aged 2–11 years, in terms of total grams (r = 0.83; *p* < 0.0001; ICC = 0.55; 95%CI 0.34–0.75), energy contribution (r = 0.79; *p* < 0.0001; ICC = 0.76; 95%CI 0.61–0.87), and weight ratio (r = 0.83; *p* < 0.0001; ICC = 0.69; 95%CI 0.50–0.83) of UPFs (Supplementary Table S7).

Consistent results emerged, especially in females, for energy ratio (r = 0.68; *p* < 0.001; ICC = 0.73; 95%CI 0.52–0.86) and weight ratio of UPFs (r = 0.75; *p* < 0.001, ICC = 0.50; 95%CI 0.25–0.75) (Supplementary Table S9).

## 4. Discussion

The present study aimed to evaluate the validity and reliability of a food frequency questionnaire specifically designed to assess the intake of food groups according to the Nova classification in Southern Italian children and adolescents. Our findings indicate that the NFFQ-Kids demonstrates good test–retest reliability for all Nova group intakes, total food, as well as relative energy and weight ratios, when compared with a 3-day food record. Additionally, acceptable validity was observed for the energy ratio of UPFs and the weight ratios of both unprocessed or minimally processed foods and UPFs.

Nowadays, a growing body of scientific literature is focusing on the ongoing UPF intake debate, which underscores its significance as a contemporary topic within nutrition and public health research. The consumption of UPFs has been associated with poor diet quality [4] and with increased risk of adverse health outcomes and mortality across the life course [5,6,7]. Clinical evidence from a randomized controlled trial has further shown that a diet rich in UPFs leads to higher ad libitum energy intake and greater body weight gain compared to an unprocessed or minimally processed diet, even when macronutrient composition and palatability are matched [24]. Importantly, these concerns extend to pediatric populations, where UPF consumption has been linked to obesity, impaired body composition, and early cardiometabolic disturbances, including dyslipidemia and altered glucose metabolism [25].

Nevertheless, few FFQs are specifically designed to gather information regarding the degree of food processing among young participants; furthermore, they fail to collect adequate data to differentiate foods based on processing, so the issue of misclassification arises. As previously mentioned, to our knowledge, this is the first FFQ specifically designed to evaluate the contribution of the Nova groups to the overall diet in South Italian children and adolescents. A similar method has been recently proposed by Oviedo-Solís and colleagues [26], who validated a semi-quantitative FFQ (SFFQ) for assessing dietary intake from each Nova group in a total of 382 Mexican children and adolescents. By comparing SFFQ with two 24 h dietary records (24DRs), the results revealed an acceptable validity for percentage energy intake from UPFs and MPFs among Mexican participants [26]. The same research group additionally validated an SFFQ to estimate energy intake for each Nova group by comparing it with two 24DRs from 226 Mexican adults, showing similar acceptable results about the energy intake from MPFs and UPFs [27]. Key differences relative to our study include the reference method (two 24 h recalls vs. a 3-day food record) and a primary focus on energy-based Nova metrics rather than combined energy and weight ratios in our assessment.

Another study validation was conducted by Fangupo and colleagues [19], who took into consideration an FFQ (EAT5 FFQ) for assessing daily energy intake and percentage of daily energy intake from each Nova group in New Zealand 5-year-old children [19]. By comparing FFQ and 3-day weight dietary record data in children from New Zealand, the results revealed an acceptable correlation for both MPFs and UPFs [19]. Compared with EAT5, our study expands the age range (2–18 y vs. a single early-childhood age specifically 5 years old), uses an estimated 3-day food record rather than a weighed record (improving feasibility for older age bands) and reports both energy and weight ratios, which better capture differences in energy density across Nova groups. In Brazil, higher UPF consumption among adolescents was associated with lower diet quality and nutrient density [28], further underscoring the need for instruments capable of capturing processing-based dietary exposures in youth. Recent efforts have also moved toward shorter screeners such as the NOVA-27 [29] or the Turkish sQ-HPF [30], which can rank individuals according to UPF intake but do not provide quantitative estimates. In contrast, the NFFQ-Kids allows both ranking and estimation of absolute contributions by processing level, a feature that enhances its utility for epidemiological and future intervention studies.

To the best of our knowledge, no FFQ has been validated in Southern Italian children and adolescents for its ability to measure the intake of food according to the level of processing as defined by the Nova classification system. Most evidence involving children and adolescents indicates that only a small proportion used 24 h recalls, while the majority relied on FFQs that were not validated for identifying UPFs [31,32].

In contrast, this study addresses the existing gap by validating a tool specifically tailored for assessing UPF consumption among children and adolescents. As such, the NFFQ-Kids offers a practical epidemiological instrument for measuring dietary processing levels, exploring their links with health outcomes, and supporting school-based surveillance or nutrition education programs.

In our study, both reliability and validity were assessed, and the present questionnaire was readjusted according to the population of interest by adding specific food items that represent food typically eaten in earlier stages of life (i.e., baby milk growth products, and baby food fruit/meat/cheese purees). Another methodological strength of our study is the use of a non-consecutive 3-day FR as the reference method. While several pediatric validation studies have relied on 24 h recalls, a 3-day record provides a more comprehensive picture of habitual intake, capturing day-to-day variability and reducing recall bias. This approach, although more demanding for participants, likely enhanced the robustness of our validity assessment compared to studies relying on single-day recalls. Another strength of our methodology is the age-based differentiation in questionnaire administration, which reflects a well-established practice in pediatric nutrition research [20] and ensures that reporting is age-appropriate. Several validation studies have adopted similar strategies, acknowledging that younger children lack the cognitive skills and memory capacity to accurately self-report, while adolescents can provide more direct but sometimes less reliable accounts of their diet [33]. Our stratified results showed that children for whom parents completed the NFFQ-Kids demonstrated higher validity and reliability coefficients (e.g., ICC up to 0.70 for UPF weight ratio validity, and 0.76–0.83 for test–retest reliability). In contrast, those who self-completed the questionnaire showed weaker correlations (fair-to-moderate validity, e.g., ICC = 0.41 for UPF weight ratio). This pattern suggests that parental proxy-report may contribute to more stable and consistent estimates [34,35], particularly for main meals, while adolescent self-report introduces variability linked to more irregular eating patterns, especially snacking, limited recall and portion-size estimation skills, and potential underreporting due to social desirability [22]. In line with this, separate analyses for parent-assisted and self-administered groups confirmed higher validity in the parent-assisted group as compared to the self-administered one.

Sex-related differences also emerged, consistent with previous literature [36,37], suggesting that girls may provide slightly more accurate dietary reports, likely due to closer attention to food choices and preparation. Overall, these findings indicate that observed differences between age groups may be partly driven by the mode of reporting rather than true dietary variation, highlighting the importance of considering both age and reporter type when interpreting FFQ data in pediatric populations.

Another limitation is that the NFFQ-Kids tended to overestimate food consumption compared to the 3-day FR, as shown by the Bland–Altman plots. This pattern is commonly reported in FFQ validation studies [38] and likely reflects multiple factors, including recall bias, the comprehensive food list, and challenges in estimating portion sizes. The plots also showed some variability in agreement across different intake levels, with larger discrepancies appearing more frequently at higher intakes for certain Nova groups. However, this systematic, slight overestimation has a limited impact when the NFFQ-Kids is used for ranking individuals or comparing groups, which is how FFQs are typically applied in epidemiological research. Second, some misclassification within the Nova system is possible because certain foods are difficult to categorize as homemade or commercially processed, regardless of their actual degree of processing. In this study, foods were classified based on their level of industrial manufacturing and ingredient composition. For example, pre-packaged items with more than five ingredients were classified as UPFs, whereas homemade or artisanal products were categorized as PFs.

Nevertheless, the problem has been addressed in earlier research in this particular scenario as well, due to the heterogeneity in food classification among different studies [39]. Moreover, while the Nova system remains the most widely used approach to classify food according to processing degree, it does not provide a direct causal link between food intake and human health, and given these constraints, the information collected should be used properly. Another limitation of the present study is the relatively small overall and subgroup sample sizes, which may reduce the statistical power and affect the generalizability of the findings.

Furthermore, the validation of the NFFQ-Kids was conducted exclusively on a sample of Southern Italian children and adolescents. Consequently, the results on validity and reliability may not be generalizable to pediatric populations of other Italian regions, where dietary habits and lifestyle factors may differ significantly.

Finally, we acknowledge the lack of formal assessment of seasonal variation in dietary intake, which could potentially influence the validity and reliability coefficients for certain food categories highly sensitive to climate (e.g., UPF beverages).

However, it is worth considering the specific Mediterranean context in which our validation was conducted. Southern Italy is characterized by the coexistence of traditional unprocessed or minimally processed foods, such as fruits, vegetables, legumes, and olive oil, with the growing availability of industrially produced UPFs. This coexistence generates a heterogeneous dietary landscape with varying levels of processing that may complicate classification and attenuate correlations with reference methods. Despite this complexity, the NFFQ-Kids achieved validity and reproducibility coefficients comparable to those obtained in more Westernized contexts such as Mexico and New Zealand, suggesting that it is sensitive enough to discriminate processing levels even in populations where traditional dietary practices remain strong.

Future studies could test this FFQ in larger and more diverse groups of Italian children, and potentially in other countries, to assess how well it performs across different settings. It would also be useful to examine whether the NFFQ-Kids can track changes in dietary patterns over time, for example, within programs aimed at reducing UPF intake. Finally, developing a digital version of the questionnaire could make it easier to administer in schools and in large population studies.

## 5. Conclusions

In recent years, there has been growing interest in UPFs and their potential health impacts, also in children and adolescents. A major challenge in this field remains the lack of dietary assessment tools specifically designed to capture data on food processing, particularly in these age groups.

In this context, the NFFQ-Kids could be a valid and reliable instrument for ranking food consumption based on processing levels in Southern Italian children and adolescents.

## Figures and Tables

**Table 1:** Participants’ characteristics.

	Whole Sample	Aged 2–11 Years	Aged 12–18 Years
N of participants (%)	73 (100.0)	43 (58.9)	30 (41.1)
Age (years)	10.6 ± 5.1	6.8 ± 2.6	16.0 ± 2.1
Males (*n*, %)	31 (42.5)	20 (46.5)	11 (36.7)
Place of residence (*n*, %)			
Rural	15 (20.5)	11 (25.6)	4 (13.3)
Urban	58 (79.5)	32 (74.4)	26 (86.7)
Food allergies or intolerances (*n*, %)	5 (6.8)	2 (4.6)	3 (10.0)
Special diet (*n*, %)	6 (8.2)	2 (4.6)	4 (13.3)
Body mass index (means, SD; kg/m^2^)	18.4 ± 4.0	16.4 ± 1.7	21.3 ± 4.4
Maternal education (*n*, %)			
Upper secondary	13 (16.4)	7 (16.3)	5 (16.7)
Bachelor’s or master’s degree	23 (31.5)	17 (39.5)	6 (20.0)
Postgraduate or higher	23 (31.5)	19 (44.2)	4 (13.3)
Missing data	15 (20.6)	0 (0.0)	15 (50.5)
Household income (*n*, %; Euros/year)			
≤25,000	11 (15.1)	8 (18.6)	3 (10.0)
25,000–40,000	21 (28.8)	17 (39.5)	4 (13.3)
≥40,000	18 (24.6)	14 (32.6)	4 (13.3)
Non responder/missing data	23 (31.5)	4 (9.3)	19 (63.4)
Maternal body mass index * (means, SD; kg/m^2^)	23.7 ± 4.6	23.7 ± 5.1	24.0 ± 3.2

* Available for 58 participants.

**Table 2:** Questionnaire validity in the whole sample (*n* = 73).

Nova Groups (g/d)	3-Day Food Records	NFFQ T0	R	*p*-Value	ICC	Lower Limit 95% CI	Upper Limit 95% CI
Unprocessed or minimally processed foods	475.8 (185.1)	677.4 (348.2)	0.54	<0.0001	0.13	0.02	0.53
Processed culinary ingredients	23.76 (11.39)	20.16 (12.87)	0.37	0.0012	0.34	0.17	0.56
Processed foods	172.1 (103.6)	279.2 (126.3)	0.29	0.014	0.10	0.01	0.59
Processed culinary ingredients + Processed foods	199.2 (104.8)	334.7 (210.2)	0.31	0.0083	0.13	0.02	0.53
Ultra-processed food	188.0 (123.2)	277.5 (221.4)	0.51	<0.0001	0.23	0.08	0.50
Total food intake	859.7 (207.4)	1254.0 (460.7)	0.49	<0.0001	0.06	0.00	0.79
**Nova groups (energy ratio)**							
Unprocessed or minimally processed foods	31.9 (10.7)	32.0 (9.4)	0.37	0.0012	0.41	0.24	0.60
Processed culinary ingredients	13.8 (6.2)	8.9 (5.8)	0.31	0.0080	0.13	0.02	0.52
Processed foods	25.6 (14.1)	30.8 (9.1)	0.31	0.0073	0.28	0.12	0.53
Processed culinary ingredients + Processed foods	39.4 (12.8)	39.7 (8.3)	0.26	0.026	0.28	0.12	0.53
Ultra-processed food	28.7 (13.1)	28.2 (10.8)	0.48	<0.0001	0.46	0.29	0.64
Total energy intake (kcal/d)	1503.0 (354.5)	2008.0 (623.2)	0.39	0.0006	0.09	0.01	0.61
**Nova groups (weight ratio)**							
Unprocessed or minimally processed foods	55.2 (14.8)	53.7 (14.5)	0.49	<0.0001	0.50	0.33	0.66
Processed culinary ingredients	2.88 (1.42)	1.74 (1.27)	0.32	0.0054	0.09	0.01	0.60
Processed foods	20.1 (11.3)	22.7 (7.7)	0.25	0.029	0.22	0.08	0.50
Processed culinary ingredients + Processed foods	22.9 (11.3)	24.4 (7.6)	0.25	0.034	0.28	0.12	0.53
Ultra-processed food	21.9 (13.1)	21.8 (13.2)	0.58	<0.0001	0.58	0.42	0.72

Data are reported as mean and standard deviation. Abbreviations: CI (confidence interval); ICC (intraclass correlation coefficients); R (Pearson correlation coefficients).

**Table 3:** Test–retest reliability (*n* = 53).

Nova Groups (g/d)	NFFQ T0	NFFQ T1	R	*p*-Value	ICC	Lower Limit 95% CI	Upper Limit 95% CI
Unprocessed or minimally processed foods	637.4 (229.0)	617.6 (248.3)	0.73	<0.0001	0.66	0.50	0.79
Processed culinary ingredients	19.3 (12.1)	18.5 (11.8)	0.42	0.0018	0.52	0.34	0.71
Processed foods	265.7 (113.8)	253.2 (110.0)	0.57	<0.0001	0.50	0.37	0.72
Processed culinary ingredients + Processed foods	285.1 (112.7)	271.8 (111.8)	0.57	<0.0001	0.54	0.35	0.72
Ultra-processed food	237.6 (165.3)	193.1 (124.4)	0.75	<0.0001	0.50	0.37	0.69
Total food intake	1160.0 (327.0)	1082.5 (323.3)	0.64	<0.0001	0.57	0.38	0.73
**Nova groups (energy ratio)**							
Unprocessed or minimally processed foods	32.6 (8.8)	34.2 (9.1)	0.50	0.0001	0.52	0.33	0.70
Processed culinary ingredients	9.2 (6.3)	9.2 (5.2)	0.54	<0.0001	0.55	0.37	0.73
Processed foods	30.7 (9.1)	32.3 (7.9)	0.60	<0.0001	0.59	0.41	0.75
Processed culinary ingredients + Processed foods	39.9 (8.2)	41.5 (7.2)	0.44	0.0010	0.45	0.25	0.66
Ultra-processed food	27.5 (11.2)	24.3 (9.4)	0.71	<0.0001	0.67	0.51	0.80
Total energy intake (kcal/d)	1903.1 (517.2)	1744.3 (393.3)	0.56	<0.0001	0.33	0.14	0.59
**Nova groups (weight ratio)**							
Unprocessed or minimally processed foods	54.9 (13.0)	56.5 (13.0)	0.76	<0.0001	0.66	0.50	0.80
Processed culinary ingredients	1.8 (1.4)	1.8 (1.2)	0.48	0.0003	0.54	0.35	0.72
Processed foods	22.9 (7.2)	23.5 (7.2)	0.64	<0.0001	0.61	0.44	0.76
Processed culinary ingredients + Processed foods	24.7 (7.0)	25.3 (7.4)	0.61	<0.0001	0.62	0.45	0.77
Ultra-processed food	20.4 (12.6)	18.1 (11.3)	0.79	<0.0001	0.67	0.51	0.80

Data are reported as mean and standard deviation. Abbreviations: CI (confidence interval); ICC (intraclass correlation coefficients); R (Pearson correlation coefficients).

## Data Availability

The data presented in this study are available on request from the corresponding author due to the ongoing nature of the study.

## Supplementary Materials

The following supporting information can be downloaded at: https://www.mdpi.com/article/10.3390/nu17233751/s1, Table S1: Questionnaire validity in children aged 2–11 years (*n* = 43); Table S2: Questionnaire validity in adolescents aged 12–18 years (*n* = 30); Table S3: Questionnaire validity in female participants (*n* = 42); Table S4: Questionnaire validity in male participants (*n* = 31); Table S5: Questionnaire validity in the parent-assisted group (*n* = 41); Table S6: Questionnaire validity in the self-administered group (*n* = 32); Table S7: Test–retest reliability in children aged 2–11 years (*n* = 39); Table S8: Test–retest reliability in adolescents aged 12–18 years (*n* = 14); Table S9: Test–retest reliability in female participants (*n* = 28); Table S10: Test–retest reliability in male participants (*n* = 25). References [40,41,42] are cited in the Supplementary Materials.

## Abbreviations

The following abbreviations are used in this manuscript:

UPFs	Ultra-processed foods
FFQ	Food frequency questionnaire
MPFs	Unprocessed or minimally processed foods
PCI	Processed culinary ingredients
PF	Processed foods
FR	Food record
CI	Confidence interval
ICC	Intraclass correlation coefficient
SFFQ	Semi-quantitative food frequency questionnaire
24DRs	24 h dietary records
