# Supplementary material for: Validity and Reliability of a Food Frequency Questionnaire (NFFQ-Kids) to Assess Food Consumption Based on the Nova Classification in Southern Italian Children and Adolescents
Source: Nutrients. 2025 Nov 28;17(23):3751. doi: 10.3390/nu17233751 (PMC12694190; doi:10.3390/nu17233751)
Supplement: Supplementary file 1 [file nutrients-17-03751-s001.zip › nutrients-3980553-supplementary.pdf]

Supplementary Materials

The NFFQ-Kids

The NFFQ-Kids is a modified version of the NFFQ for the Italian adult population [1] and was adapted to collect dietary intakes and the level of processing of the diet of Italian children aged ≥2 and ≤18 years over the past 3 months. The NFFQ-Kids consists of 107 items, which are gathered in 9 food groups: 'fruit and nuts', 'vegetables and legumes', 'cereals and tubers', 'meat and fish', 'milk, dairy products, and eggs', 'oils, fats, and seasonings', 'sweets and sweeteners', 'beverages', 'other', and an additional table to note frequently consumed items not present in the previous sections. However, to better capture nutritional information in children and adolescents, additional items were integrated into the original list of items of the NFFQ. 'Fruit and nuts', 'vegetables and legumes', and 'meat and fish' sections were added of both homemade and industrial preparations, homogenised and/or freeze-dried. The 'Cereals and tubers' section was added for industrial freeze-dried cereal products. 'Milk, dairy products, and eggs' section was added for dry-powdered and ready-to-feed milk formulas, and artisanal and freeze-dried cheeses. 'Oils, fats, and seasonings' were divided into separate items: vegetable oils, olive oil, and extra-virgin olive oil. In the 'beverages' section, beer, wine, and other alcoholic beverages were excluded for participants aged 2 to 11 years. The degree of food processing of all food items and ingredients was evaluated using the Nova classification system [2], by mapping and grouping all FFQ items into the four Nova categories. All items recognisable as natural and fresh food that can only be subjected to minimal processing (i.e., washing, milling, chilling, freezing, or vacuum-packing) were classified as MPFs. Cooking ingredients not eatable as such (i.e., oils and fats, salt, and sugars) were classified as PCI. Processed foods, generally the result of the addition of PCI to MPF, were classified as PF. Given their frequent combined use in various preparations, PCI and PF were grouped together in the analysis. Finally, all food items identifiable as complex industrial formulations, composed of 5 or more ingredients, possibly added with preservatives and additives, visually and economically attractive, and highly palatable were categorised as UPFs. The frequency of food consumption could be reported by choosing among ten options: (1) "never or less than once a month", (2) "one-three times per month", (3) "once a week", (4) "two times per week", (5) "three times per week", (6) "four times per week", (7) "five times per week", (8) "six times per week", (9) "every day" and (10) "if every day, how many times per day?". Participants could indicate their usual portion size by selecting one out of six options, from 0.5 to 3 portions. As for the latter, reference portions were established using the "Quantitative standards for portions" of the Italian Society of Human Nutrition [3] or in the Dietary Guidelines for the Italian population [4]. If not available, the serving size displayed on the food label of products currently on the market was used as a reference portion (e.g., packaged instant rice, soups or noodles, plant-based meat substitutes, health and slimming products including meal replacement shakes and powders).

Table S1. Questionnaire validity in children aged 2–11 years (n = 43).

**Nova groups (g/d)**	**3-day food records**	**NFFQ T0**	**R**	**p-value**	**ICC**	**Lower limit 95% CI**	**Upper limit 95% CI**
Unprocessed or minimally processed foods	489.8 (168.2)	637.0 (238.1)	0.42	0.0057	0.15	0.02	0.63
Processed culinary ingredients	25.9 (12.0)	18.7 (12.1)	0.37	0.0014	0.26	0.07	0.60
Processed foods	152.6 (80.8)	253.0 (118.5)	0.31	0.040	0.09	0.00	0.77
Processed culinary ingredients + Processed foods	178.5 (83.1)	271.7 (116.4)	0.32	0.035	0.15	0.02	0.64
Ultra-processed food	171.7 (109.5)	232.9 (173.5)	0.56	<.0001	0.42	0.21	0.66
Total food intake	840.2 (211.0)	1142.0 (331.4)	0.43	0.0036	0.04	0.00	0.99
**Nova groups (energy ratio)**							
Unprocessed or minimally processed foods	32.3 (9.2)	33.6 (8.9)	0.37	0.014	0.43	0.22	0.67
Processed culinary ingredients	15.6 (6.3)	9.5 (6.6)	0.28	0.072	0.07	0.00	0.90
Processed foods	24.1 (13.2)	29.5 (9.9)	0.37	0.015	0.37	0.17	0.64
Processed culinary ingredients + Processed foods	39.7 (11.9)	39.0 (8.5)	0.36	0.019	0.39	0.18	0.65
Ultra-processed food	28.7 (13.1)	28.2 (10.8)	0.48	<.0001	0.50	0.29	0.71
Total energy intake (kcal/d)	1454.0 (339.2)	1794.0 (382.6)	0.19	0.22	0.09	0.00	0.80
**Nova groups (weight ratio)**							
Unprocessed or minimally processed foods	58.2 (12.1)	55.7 (13.8)	0.42	0.0047	0.47	0.26	0.69
Processed culinary ingredients	3.23 (1.55)	1.83 (1.49)	0.32	0.036	0.06	0.00	0.94
Processed foods	18.4 (9.5)	22.2 (8.3)	0.31	0.045	0.24	0.06	0.60
Processed culinary ingredients + Processed foods	21.6 (9.7)	24.1 (8.1)	0.28	0.068	0.39	0.18	0.65
Ultra-processed food	20.1 (11.5)	20.3 (13.3)	0.67	<.0001	0.70	0.53	0.83

Data are reported as mean and standard deviation. Abbreviations: CI (confidence interval); ICC (intraclass correlation coefficients); R (Pearson correlation coefficients).

Table S2. Questionnaire validity in adolescents aged 12–18 years (n = 30).

Nova groups (g/d)	3-day food records	NFFQ T0	R	p-value	ICC	Lower limit 95% CI	Upper limit 95% CI
Unprocessed or minimally processed foods	455.8 (208.2)	735.3 (461.5)	0.66	<.0001	0.11	0.00	0.81
Processed culinary ingredients	20.6 (9.8)	22.2 (13.8)	0.52	0.0032	0.46	0.22	0.73
Processed foods	200.0 (125.9)	316.7 (129.5)	0.21	0.26	0.02	0.00	1.00
Processed culinary ingredients + Processed foods	220.6 (126.3)	339.0 (133.9)	0.23	0.22	0.03	0.00	1.00
Ultra-processed food	211.3 (139.0)	341.4 (266.3)	0.40	0.027	0.05	0.00	0.99
Total food intake	887.8 (202.3)	1416.0 (567.6)	0.55	0.0015	-	-	-
**Nova groups (energy ratio)**							
Unprocessed or minimally processed foods	31.4 (12.7)	29.6 (9.6)	0.38	0.036	0.37	0.13	0.69
Processed culinary ingredients	11.3 (5.2)	8.1 (4.3)	0.33	0.072	0.17	0.02	0.71
Processed foods	27.7 (15.2)	32.7 (7.7)	0.24	0.20	0.12	0.01	0.79
Processed culinary ingredients + Processed foods	39.0 (14.2)	40.8 (8.1)	0.17	0.38	0.16	0.01	0.71
Ultra-processed food	29.6 (14.5)	29.6 (10.0)	0.47	0.0083	0.40	0.16	0.70
Total energy intake (kcal/d)	1574.0 (369.6)	2315.0 (766.2)	0.44	0.014	-	-	-
**Nova groups (weight ratio)**							
Unprocessed or minimally processed foods	50.8 (17.3)	51.0 (15.2)	0.52	0.0035	0.48	0.23	0.73
Processed culinary ingredients	2.37 (1.02)	1.61 (0.89)	0.29	0.13	0.09	0.00	0.89
Processed foods	22.4 (13.4)	23.3 (6.9)	0.24	0.21	0.19	0.02	0.69
Processed culinary ingredients + Processed foods	24.8 (13.3)	24.9 (7.0)	0.26	0.17	0.16	0.01	0.71
Ultra-processed food	24.4 (14.9)	24.1 (12.9)	0.41	0.026	0.41	0.17	0.71

Data are reported as mean and standard deviation. Abbreviations: CI (confidence interval); ICC (intraclass correlation coefficients); R (Pearson correlation coefficients).

Table S3. Questionnaire validity in female participants (n = 42).

Nova groups (g/d)	3-day food records	NFFQ T0	R	p-value	ICC	Lower limit 95% CI	Upper limit 95% CI
Unprocessed or minimally processed foods	456.8 (150.9)	676.5 (366.6)	0.44	0.0039	0.03	0.00	1.00
Processed culinary ingredients	25.0 (12.3)	21.0 (14.5)	0.46	0.0020	0.41	0.20	0.66
Processed foods	161.6 (96.9)	274.2 (130.3)	0.29	0.062	0.00	-	-
Processed culinary ingredients + Processed foods	186.6 (97.7)	295.2 (131.4)	0.28	0.072	0.03	0.00	1.00
Ultra-processed food	178.1 (128.5)	275.1 (262.8)	0.62	<.0001	0.16	0.02	0.63
Total food intake	821.5 (173.1)	1246.8 (493.5)	0.43	0.0048	0.00	-	-
**Nova groups (energy ratio)**							
Unprocessed or minimally processed foods	32.5 (11.9)	32.6 (10.6)	0.46	0.0023	0.48	0.26	0.70
Processed culinary ingredients	15.1 (6.5)	5.1 (6.5)	0.29	0.059	0.13	0.01	0.68
Processed foods	25.3 (15.0)	30.6 (9.7)	0.29	0.060	0.25	0.07	0.60
Processed culinary ingredients + Processed foods	40.3 (13.5)	40.2 (8.2)	0.19	0.22	0.24	0.06	0.60
Ultra-processed food	27.1 (13.3)	27.2 (11.1)	0.60	<.0001	0.47	0.25	0.69
Total energy intake (kcal/d)	1440.0 (340.6)	1945.8 (605.1)	0.31	0.044	0.01	0.00	1.00
**Nova groups (weight ratio)**							
Unprocessed or minimally processed foods	56.1 (15.1)	54.5 (15.9)	0.48	0.0012	0.47	0.26	0.70
Processed culinary ingredients	3.1 (1.4)	1.9 (1.5)	0.32	0.036	0.14	0.01	0.66
Processed foods	20.1 (12.5)	22.4 (8.1)	0.29	0.060	0.27	0.08	0.60
Processed culinary ingredients + Processed foods	23.2 (12.6)	24.3 (8.1)	0.30	0.053	0.24	0.06	0.60
Ultra-processed food	20.6 (12.4)	21.2 (14.4)	0.59	<.0001	0.54	0.33	0.70

Data are reported as mean and standard deviation. Abbreviations: CI (confidence interval); ICC (intraclass correlation coefficients); R (Pearson correlation coefficients).

Table S4. Questionnaire validity in male participants (n = 31).

Nova groups (g/d)	3-day food records	NFFQ T0	R	p-value	ICC	Lower limit 95% CI	Upper limit 95% CI
Unprocessed or minimally processed foods	501.7 (223.3)	678.5 (327.5)	0.64	0.0001	0.26	0.06	0.66
Processed culinary ingredients	22.1 (10.0)	19.0 (10.3)	0.22	0.24	0.14	0.01	0.73
Processed foods	186.3 (112.2)	286.0 (122.4)	0.27	0.15	0.23	0.05	0.66
Processed culinary ingredients + Processed foods	208.3 (113.2)	304.9 (123.8)	0.30	0.10	0.25	0.06	0.66
Ultra-processed food	201.3 (116.3)	280.8 (152.5)	0.37	0.043	0.41	0.17	0.70
Total food intake	911.4 (239.7)	1264.3 (420.0)	0.53	0.0023	0.13	0.01	0.76
**Nova groups (energy ratio)**							
Unprocessed or minimally processed foods	31.0 (8.8)	31.2 (7.4)	0.19	0.31	0.52	0.28	0.75
Processed culinary ingredients	12.2 (5.5)	7.9 (4.1)	0.34	0.059	0.00	-	-
Processed foods	26.0 (12.9)	31.2 (8.4)	0.37	0.039	0.17	0.02	0.70
Processed culinary ingredients + Processed foods	38.1 (11.9)	39.1 (8.6)	0.35	0.056	0.35	0.12	0.68
Ultra-processed food	30.8 (12.6)	29.6 (10.5)	0.36	0.044	0.73	0.54	0.86
Total energy intake (kcal/d)	1589.2 (360.2)	2092.7 (647.4)	0.54	0.0019	0.16	0.01	0.71
**Nova groups (weight ratio)**							
Unprocessed or minimally processed foods	53.9 (14.4)	52.8 (12.4)	0.50	0.0042	0.22	0.04	0.66
Processed culinary ingredients	2.6 (1.4)	1.6 (0.8)	0.36	0.052	0.03	0.00	1.00
Processed foods	19.9 (9.7)	23.0 (7.2)	0.21	0.25	0.31	0.06	0.67
Processed culinary ingredients + Processed foods	22.5 (9.6)	24.6 (7.1)	0.20	0.29	0.35	0.12	0.68
Ultra-processed food	23.6 (13.9)	22.6 (11.5)	0.53	0.0002	0.31	0.09	0.67

Data are reported as mean and standard deviation. Abbreviations: CI (confidence interval); ICC (intraclass correlation coefficients); R (Pearson correlation coefficients).

Table S5. Questionnaire validity in the parent-assisted group (n = 41).

Nova groups (g/d)	3-day food records	NFFQ T0	R	p-value	ICC	Lower limit 95% CI	Upper limit 95% CI
Unprocessed or minimally processed foods	451.1 (158.4)	614.2 (234.8)	0.49	0.0011	0.16	0.02	0.63
Processed culinary ingredients	24.8 (11.1)	18.5 (12.2)	0.35	0.024	0.28	0.09	0.61
Processed foods	148.0 (81.4)	240.5 (109.4)	0.34	0.029	0.14	0.01	0.66
Processed culinary ingredients + Processed foods	172.7 (81.8)	259.0 (107.6)	0.33	0.034	0.19	0.03	0.61
Ultra-processed food	165.6 (109.1)	228.5 (169.8)	0.54	0.0002	0.41	0.20	0.67
Total food intake	788.5 (164.1)	1101.7 (307.3)	0.36	0.022	-	-	-
**Nova groups (energy ratio)**							
Unprocessed or minimally processed foods	31.7 (9.6)	33.7 (9.1)	0.44	0.0035	0.46	0.25	0.70
Processed culinary ingredients	15.6 (6.4)	9.6 (6.7)	0.29	0.066	0.08	0.00	0.83
Processed foods	24.5 (14.1)	29.0 (9.9)	0.38	0.013	0.39	0.18	0.66
Processed culinary ingredients + Processed foods	40.1 (12.6)	38.5 (8.5)	0.39	0.011	0.40	0.19	0.66
Ultra-processed food	28.2 (12.0)	27.7 (12.2)	0.57	0.0001	0.58	0.38	0.76
Total energy intake (kcal/d)	1389.1 (255.1)	1770.1 (374.3)	0.067	0.68	-	-	-
**Nova groups (weight ratio)**							
Unprocessed or minimally processed foods	55.5 (14.1)	56.9 (13.8)	0.55	0.0002	0.57	0.36	0.75
Processed culinary ingredients	1.9 (1.5)	3.3 (1.6)	0.29	0.065	0.03	0.00	1.00
Processed foods	21.9 (8.0)	19.2 (11.2)	0.39	0.012	0.35	0.14	0.63
Processed culinary ingredients + Processed foods	23.8 (7.8)	22.5 (11.4)	0.37	0.017	0.40	0.19	0.66
Ultra-processed food	20.7 (13.7)	20.6 (12.2)	0.72	<.0001	0.73	0.57	0.85

Data are reported as mean and standard deviation. Abbreviations: CI (confidence interval); ICC (intraclass correlation coefficients); R (Pearson correlation coefficients).

Table S6. Questionnaire validity in the self-administered group (n = 32).

Nova groups (g/d)	3-day food records	NFFQ T0	R	p-value	ICC	Lower limit 95% CI	Upper limit 95% CI
Unprocessed or minimally processed foods	507.6 (212.9)	758.3 (445.3)	0.54	0.0014	0.08	0.00	0.92
Processed culinary ingredients	22.5 (11.8)	22.3 (13.6)	0.50	0.0035	0.40	0.16	0.69
Processed foods	202.9 (121.0)	328.7 (130.7)	0.15	0.43	-	-	-
Processed culinary ingredients + Processed foods	225.4 (112.7)	351.0 (133.7)	0.18	0.31	-	-	-
Ultra-processed food	217.9 (135.0)	340.3 (263.5)	0.38	0.030	0.05	0.00	0.98
Total food intake	950.9 (223.3)	1449.6 (548.6)	0.49	0.0046	-	-	-
**Nova groups (energy ratio)**							
Unprocessed or minimally processed foods	32.1 (12.0)	29.8 (9.4)	0.34	0.057	0.34	0.12	0.67
Processed culinary ingredients	11.7 (5.3)	8.1 (4.3)	0.34	0.056	0.13	0.01	0.76
Processed foods	26.9 (14.1)	33.2 (7.6)	0.24	0.18	0.10	0.00	0.84
Processed culinary ingredients + Processed foods	38.6 (13.3)	41.3 (8.0)	0.14	0.45	0.14	0.01	0.74
Ultra-processed food	29.3 (14.5)	28.9 (8.9)	0.37	0.039	0.29	0.08	0.66
Total energy intake (kcal/d)	1649.8 (410.5)	2313.3 (741.8)	0.36	0.041	-	-	-
**Nova groups (weight ratio)**							
Unprocessed or minimally processed foods	51.5 (14.8)	52.9 (16.0)	0.39	0.026	0.40	0.16	0.69
Processed culinary ingredients	1.6 (0.9)	2.3 (1.0)	0.39	0.029	0.15	0.01	0.71
Processed foods	23.7 (7.3)	21.2 (11.6)	0.15	0.40	0.05	0.00	0.98
Processed culinary ingredients + Processed foods	25.3 (7.4)	23.6 (11.5)	0.12	0.51	0.14	0.01	0.74
Ultra-processed food	23.2 (12.5)	23.5 (14.2)	0.36	0.045	0.38	0.15	0.68

Data are reported as mean and standard deviation. Abbreviations: CI (confidence interval); ICC (intraclass correlation coefficients); R (Pearson correlation coefficients).

Table S7. Test–retest reliability in children aged 2–11 years (n = 39).

Nova groups (g/d)	NFFQ T0	NFFQ T1	R	p-value	ICC	Lower limit 95% CI	Upper limit 95% CI
Unprocessed or minimally processed foods	625.0 (232.6)	606.8 (254.3)	0.73	<.0001	0.65	0.46	0.81
Processed culinary ingredients	18.9 (12.6)	19.4 (11.9)	0.46	<.0001	0.62	0.42	0.79
Processed foods	252.3 (116.8)	259.3 (121.2)	0.72	<.0001	0.69	0.50	0.82
Processed culinary ingredients + Processed foods	271.2 (114.6)	278.7 (112.4)	0.74	<.0001	0.68	0.50	0.82
Ultra-processed food	232.4 (180.4)	191.4 (136.4)	0.83	<.0001	0.55	0.34	0.75
Total food intake	1128.6 (332.5)	1076.9 (332.7)	0.72	<.0001	0.65	0.46	0.81
**Nova groups (energy ratio)**							
Unprocessed or minimally processed foods	33.7 (9.2)	33.5 (9.3)	0.61	<.0001	0.67	0.48	0.81
Processed culinary ingredients	9.5 (6.9)	9.9 (5.3)	0.55	0.0002	0.54	0.33	0.74
Processed foods	29.4 (9.7)	32.0 (8.5)	0.73	<.0001	0.67	0.48	0.81
Processed culinary ingredients + Processed foods	38.9 (8.4)	41.9 (7.3)	0.55	0.0003	0.51	0.29	0.73
Ultra-processed food	27.4 (11.6)	24.6 (10.2)	0.79	<.0001	0.76	0.61	0.87
Total energy intake (kcal/d)	1801.4 (394.9)	1742.6 (406.7)	0.68	<.0001	0.72	0.54	0.84
**Nova groups (weight ratio)**							
Unprocessed or minimally processed foods	55.4 (14.0)	55.8 (14.2)	0.80	<.0001	0.70	0.52	0.83
Processed culinary ingredients	1.9 (1.5)	2.0 (1.2)	0.51	0.0009	0.54	0.33	0.74
Processed foods	22.4 (7.9)	24.2 (8.1)	0.68	<.0001	0.65	0.45	0.80
Processed culinary ingredients + Processed foods	24.3 (7.7)	26.1 (8.2)	0.66	<.0001	0.65	0.46	0.80
Ultra-processed food	20.4 (13.8)	18.0 (12.3)	0.83	<.0001	0.69	0.50	0.83

Data are reported as mean and standard deviation. Abbreviations: CI (confidence interval); ICC (intraclass correlation coefficients); R (Pearson correlation coefficients).

Table S8. Test–retest reliability in adolescents aged 12–18 years (n = 14).

Nova groups (g/d)	NFFQ T0	NFFQ T1	R	p-value	ICC	Lower limit 95% CI	Upper limit 95% CI
Unprocessed or minimally processed foods	671.7 (223.3)	647.8 (237.3)	0.68	0.0076	0.68	0.36	0.88
Processed culinary ingredients	20.4 (11.0)	16.0 (11.3)	0.39	0.017	0.18	0.01	0.87
Processed foods	303.2 (99.4)	236.3 (70.9)	0.06	0.84	-	-	-
Processed culinary ingredients + Processed foods	323.7 (101.0)	252.3 (75.2)	0.13	0.65	-	-	-
Ultra-processed food	252.0 (118.0)	197.8 (86.5)	0.51	0.061	0.17	0.01	0.88
Total food intake	1247.4 (305.5)	1097.9 (306.9)	0.32	0.27	0.27	0.03	0.81
**Nova groups (energy ratio)**							
Unprocessed or minimally processed foods	29.4 (6.6)	36.0 (8.6)	0.44	0.12	-	-	-
Processed culinary ingredients	8.2 (4.1)	7.3 (4.7)	0.67	0.0087	0.54	0.21	0.84
Processed foods	34.4 (6.0)	33.0 (6.1)	-0.13	0.65	0.02	0.00	1.00
Processed culinary ingredients + Processed foods	42.6 (7.1)	40.3 (6.8)	0.27	0.34	0.31	0.04	0.80
Ultra-processed food	28.0 (10.6)	23.7 (6.7)	0.32	0.26	0.20	0.01	0.85
Total energy intake (kcal/d)	2186.1 (704.8)	1749.1 (367.8)	0.086	0.77	-	-	-
**Nova groups (weight ratio)**							
Unprocessed or minimally processed foods	53.5 (9.6)	58.4 (9.1)	0.72	0.0035	0.46	0.14	0.82
Processed culinary ingredients	1.7 (0.8)	1.4 (1.0)	0.49	0.075	0.45	0.13	0.82
Processed foods	24.2 (4.7)	21.7 (3.5)	0.50	0.069	0.27	0.03	0.81
Processed culinary ingredients + Processed foods	25.8 (4.6)	23.1 (3.7)	0.55	0.041	0.30	0.04	0.81
Ultra-processed food	20.6 (9.0)	18.5 (8.3)	0.63	0.015	0.54	0.21	0.84

Data are reported as mean and standard deviation. Abbreviations: CI (confidence interval); ICC (intraclass correlation coefficients); R (Pearson correlation coefficients).

Table S9. Test–retest reliability in female participants (n = 28).

Nova groups (g/d)	NFFQ T0	NFFQ T1	R	p-value	ICC	Lower limit 95% CI	Upper limit 95% CI
Unprocessed or minimally processed foods	632.0 (242.3)	615.2 (233.9)	0.63	0.0003	0.61	0.37	0.81
Processed culinary ingredients	21.1 (14.4)	19.6 (13.0)	0.64	<.0001	0.65	0.42	0.83
Processed foods	253.6 (122.3)	246.3 (118.0)	0.68	<.0001	0.65	0.42	0.83
Processed culinary ingredients + Processed foods	274.8 (120.6)	265.9 (119.7)	0.72	<.0001	0.65	0.42	0.82
Ultra-processed food	208.1 (169.8)	163.1 (96.1)	0.81	<.0001	0.36	0.12	0.69
Total food intake	1114.9 (322.6)	1044.2 (292.0)	0.63	0.0003	0.61	0.38	0.81
**Nova groups (energy ratio)**							
Unprocessed or minimally processed foods	33.1 (10.5)	34.8 (9.9)	0.60	0.0006	0.63	0.40	0.82
Processed culinary ingredients	10.7 (7.6)	9.9 (5.9)	0.63	0.0003	0.61	0.37	0.81
Processed foods	30.4 (10.5)	32.0 (9.0)	0.63	0.0003	0.63	0.39	0.81
Processed culinary ingredients + Processed foods	41.1 (8.4)	41.9 (8.2)	0.43	0.023	0.51	0.26	0.76
Ultra-processed food	25.8 (11.2)	23.3 (10.3)	0.68	<.0001	0.73	0.52	0.86
Total energy intake (kcal/d)	1787.1 (412.8)	1738.7 (412.7)	0.68	<.0001	0.64	0.41	0.82
**Nova groups (weight ratio)**							
Unprocessed or minimally processed foods	56.6 (14.5)	58.3 (14.2)	0.76	<.0001	0.65	0.43	0.83
Processed culinary ingredients	2.1 (1.7)	2.0 (1.4)	0.58	0.0013	0.58	0.33	0.79
Processed foods	22.6 (7.9)	23.3 (7.5)	0.67	0.0001	0.67	0.44	0.83
Processed culinary ingredients + Processed foods	24.7 (7.7)	25.2 (7.8)	0.67	<.0001	0.69	0.47	0.85
Ultra-processed food	18.7 (13.1)	16.4 (10.8)	0.75	<.0001	0.50	0.25	0.75

Data are reported as mean and standard deviation. Abbreviations: CI (confidence interval); ICC (intraclass correlation coefficients); R (Pearson correlation coefficients).

Table S10. Test–retest reliability in male participants (n = 25).

Nova groups (g/d)	NFFQ T0	NFFQ T1	R	p-value	ICC	Lower limit 95% CI	Upper limit 95% CI
Unprocessed or minimally processed foods	643.4 (217.9)	620.3 (268.4)	0.81	<.0001	0.71	0.49	0.86
Processed culinary ingredients	17.3 (8.8)	17.4 (10.5)	0.06	0.77	0.21	0.03	0.72
Processed foods	279.3 (104.2)	261.0 (102.1)	0.38	0.061	0.39	0.14	0.72
Processed culinary ingredients + Processed foods	296.6 (104.3)	278.3 (104.3)	0.32	0.11	0.37	0.12	0.72
Ultra-processed food	270.6 (157.0)	226.7 (144.7)	0.65	0.0004	0.60	0.34	0.81
Total food intake	1210.5 (331.0)	1125.3 (356.3)	0.55	0.0045	0.51	0.25	0.77
**Nova groups (energy ratio)**							
Unprocessed or minimally processed foods	31.9 (6.5)	33.5 (8.2)	0.33	0.11	0.28	0.06	0.70
Processed culinary ingredients	7.5 (3.9)	8.4 (4.3)	0.40	0.047	0.31	0.08	0.70
Processed foods	31.1 (7.5)	32.6 (6.6)	0.51	0.0096	0.51	0.25	0.77
Processed culinary ingredients + Processed foods	38.6 (7.9)	41.0 (5.9)	0.37	0.071	0.34	0.10	0.71
Ultra-processed food	29.5 (11.1)	25.5 (8.3)	0.71	<.0001	0.59	0.33	0.81
Total energy intake (kcal/d)	2032.9 (595.4)	1750.6 (378.8)	0.50	0.012	0.08	0.00	0.93
**Nova groups (weight ratio)**							
Unprocessed or minimally processed foods	52.9 (11.0)	54.5 (11.5)	0.68	0.0002	0.66	0.43	0.84
Processed culinary ingredients	1.5 (0.8)	1.6 (11.5)	0.30	0.15	0.32	0.09	0.70
Processed foods	23.1 (6.4)	23.8 (7.0)	0.57	0.0028	0.54	0.27	0.78
Processed culinary ingredients + Processed foods	24.7 (6.3)	25.4 (7.1)	0.54	0.0052	0.52	0.25	0.77
Ultra-processed food	22.4 (12.1)	20.1 (11.7)	0.80	<.0001	0.84	0.69	0.92

Data are reported as mean and standard deviation. Abbreviations: CI (confidence interval); ICC (intraclass correlation coefficients); R (Pearson correlation coefficients).

References

1. Dinu, M.; Bonaccio, M.; Martini, D.; Madarena, M.P.; Vitale, M.; Pagliai, G.; Esposito, S.; Ferraris, C.; Guglielmetti, M.; Rosi, A.; Angelino, D. Reproducibility and validity of a food-frequency questionnaire (NFFQ) to assess food consumption based on the NOVA classification in adults. Int. J. Food Sci. Nutr. 2021, 72, 861–869, doi:10.1080/09637486.2021.1880552.
2. Monteiro, C.A.; Cannon, G.; Moubarac, J.-C.; Levy, R.B.; Louzada, M.L.C.; Jaime, P.C. The UN Decade of Nutrition, the NOVA food classification and the trouble with ultra-processing. Public Health Nutr. 2018, 21, 5–17, doi:10.1017/S1368980017000234.
3. Scalfi, L.; Censi, L.; Marra, M.; Maffeis, C.; Pecoraro, P.; Polito, A.; Strata, A.; Tagliabue, A. LARN Livelli di Assunzione di Riferimento di Nutrienti ed energia per la popolazione italiana - IV Revisione. SINU 2014.
4. CREA Linee guida per una sana alimentazione; CREA, R. 2018., Ed.; 2019; pp. 1–229;.
